# Pneumonitis

**Published:** 1871-02

**Authors:** 


PNEUMONITIS.—So far as published statistics can be relied on, the mortality of pneumonitis varies, under different modes of treatment, from one in two and a-half (by Rasori's) to one in thirty-two (by Bennett's). In England seventeen per cent. of the deaths are said to be caused by pneumonia, and in this country its death-rate is thought to be still higher.

